# Life on the wall: the diversity and activity of microbes on 13th – century AD. Lan Na mural painting

**DOI:** 10.3389/fmicb.2023.1220901

**Published:** 2023-09-15

**Authors:** Chakriya Sansupa, Nattaphon Suphaphimol, Paradha Nonthijun, Teewararat Ronsuek, Saranphong Yimklan, Natthawat Semakul, Thapakorn Khrueraya, Nakarin Suwannarach, Witoon Purahong, Terd Disayathanoowat

**Affiliations:** ^1^Department of Biology, Faculty of Science, Chiang Mai University, Chiang Mai, Thailand; ^2^Department of Chemistry, Faculty of Science, Chiang Mai University, Chiang Mai, Thailand; ^3^Faculty of Liberal Arts, Maejo University, Chiang Mai, Thailand; ^4^Research Center of Microbial Diversity and Sustainable Utilization, Chiang Mai University, Chiang Mai, Thailand; ^5^Department of Soil Ecology, UFZ-Helmholtz Centre for Environmental Research, Halle (Saale), Germany

**Keywords:** biodeterioration of wall painting, deterioration of cultural heritage, microbiome associated with mural paintings, crystal formation on painting, biomineralization

## Abstract

Diverse microorganisms from the three domains of life (Archaea, Bacteria, and Eukaryota) cause deterioration in mural paintings worldwide; however, few studies have simultaneously targeted these three domains. This study aims to survey the microbiome and its potential for biodeterioration on unpreserved Lan Na mural paintings in Sean Khan temple, Chiang Mai, Thailand. The overview of the archaeal, bacterial, and fungal communities was reported by Illumina sequencing, whereas the potential for deterioration was revealed by culturable techniques and a literature search. The abundant microbes reported in this study were also found in other ancient mural paintings worldwide. *Halococcus*, a salt-tolerant archaeon, as well as the eubacterial genus *Crossiella* dominated the prokaryotic community. On the other hand, the main fungal group was the genus *Candida* (*Ascomycota*). However, a low number of fungi and bacteria were isolated. Most of the isolates showed the ability to survive in the drought conditions of mural paintings but could not perform discoloration activities. The deterioration activity mainly affected calcium compounds, which are the main components of painting substrates. *Aspergillus* and several bacterial isolates could dissolve calcium compounds, but only *Trichaptum* species could induce crystal formation. These results suggest that deterioration of painting substrate should be taken into consideration in addition to deterioration of color in mural paintings. For the Lan Na painting in Sean Khan temple, the plaster is the prime target for biodeterioration, and thus we suggest that the preservation effort should focus on this component of the mural painting.

## 1. Introduction

Paint is one of the world’s oldest manmade products, with roots dating back to prehistoric times (Ravikumar, 2012). Paintings, like murals in a historical building heritage, are crucial landmarks that reflect and contribute to the long-term development of a region or country. In addition, it is linked to a sense of self-identity and is frequently exploited by the tourism industry. As past studies have demonstrated that the decline of mural paintings can impact social and economic systems, it is crucial to conserve these artworks for historical research as well as to ensure the continued social and economic stability of these systems (Silverman and Ruggles, 2007).

The deterioration of mural paintings can be influenced by several threats. These include intrinsic changes in the substrates’ physicochemical properties as well as extrinsic factors like atmospheric conditions and biological activities (Tiano, 2002; Dahmani et al., 2020). Atmospheric conditions and environmental factors, such as humidity, temperature, pH, and light, may cause structural or aesthetic damage to the paintings (Sterflinger, 2000; Warscheid and Braams, 2000; Rosling et al., 2009). Furthermore, as paintings contain a wide variety of organic and inorganic materials, they can be exploited by a wide range of microbial species. If the environmental conditions are favorable, many different types of microbes can colonize the paintings and cause damage to the artwork (Ciferri, 1999).

Diverse microorganisms, including archaea, bacteria and fungi, contribute to the destruction of cultural assets, especially those that contain elements required for their metabolism (Nuhoglu et al., 2006; Wiktor et al., 2009; Ettenauer et al., 2014; Karaca et al., 2015; Mihajlovski et al., 2017). Few studies have investigated simultaneously these three domains. Discoloration of paint and structural deconstruction of the painting layer are major issues caused by these organisms, leading to the deterioration of the art work (Ciferri, 1999). Microorganisms can utilize the organic compounds in the painting layers and produce enzymes or organic acids that decolorized and dissolved the compounds in the painting. Calcium compounds, for example, can be dissolved by many microorganisms (Rölleke et al., 1996; Rosado et al., 2013; Suphaphimol et al., 2022). Biomineralization is the process by which living organisms influence the precipitation of mineral materials (Skinner and Ehrlich, 2014). The formation of salt or calcium compound can also lead to the deconstruction of the painting’s layer, as it could increase the pressure on the mural materials and crack the artwork (Pérez-Alonso et al., 2004; Piñar and Sterflinger, 2009; Čukovska et al., 2017; Suphaphimol et al., 2022). Due to the ability of microorganisms to cause mural deterioration, as described above, it is of utmost importance to observe and investigate the possible threats that these organisms pose to a mural painting. As a result, we can determine the deterioration risk of the mural paintings and suggest promising ways to preserve them.

Lan Na culture is the unique culture of the northern Thailand and their neighborhood area because this kingdom is considered as the melting pot of big cultures especially Siamese (Central part of Thailand), Chinese and Burmese (Myanmar). Lan Na heritage is unique for its cuisine, architecture, clothing and mural paintings for over 700 years. One of the remained pieces of evidence of the last dynasty named Tipjakara (1732–1943) (Penth, 2001) and the biodeterioration of their mural painting was monitored (Suphaphimol et al., 2022). Only two mural painting sites from the first dynasty named Mungrai (1296–1578) (Penth, 2001) were found, one is a well preserved mural painting in the tunnel of Wat Umong, and another the unpreserved mural painting in Wat Sean Khan.

In this study, we investigated the microbial communities from the three domains of life associated with the mural painting in Sean Khan temple and determined the biodeterioration potentials of these microbes. Specifically, we identified archaea, bacteria and fungi using high-throughput sequencing and isolated bacteria and fungi using traditional culture methods. We then subjected the microbial isolates to deterioration tests, including discoloration, solubilization of calcium compounds, and crystal formation. Our study aimed to answer the following questions: (i) What microbial taxa dominate the mural paintings? (ii) Are any of these taxa capable of deteriorating the paintings? and (iii) How do they cause deterioration? To our knowledge, this study is among the few to reveal information about the microbes associated with mural paintings in Lan Na culture, and it is the first to contribute such information about mural art in the Mungrai dynasty period.

## 2. Materials and methods

### 2.1. Site description

**Figure 1:**
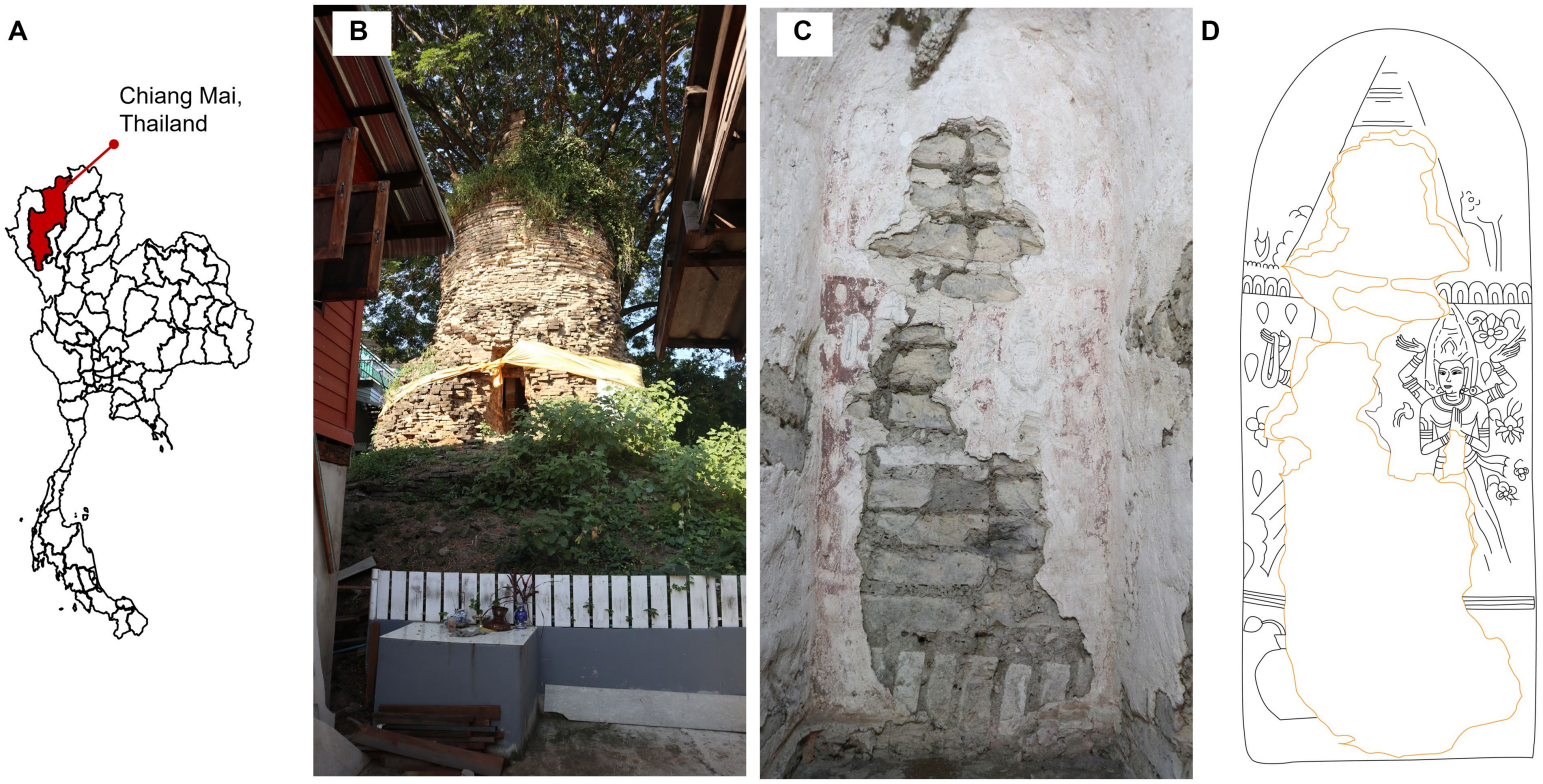
Study site. **(A)** Study site location, **(B)** landscape outside the pagoda, **(C)** overview of mural painting on the wall inside the pagoda tunnel and **(D)** skatch of the mural painting.The pagoda and mural photoes were taken by Thapakorn Khrueraya.

This study was carried out at Sean Khan temple, Chiang Mai, northern Thailand (18°47′ 48′′ N, 99° 59′ 4′′ E). The climatic data from 2008–2022 showed the minimum and maximum ranging from 9°C in December to 42.2°C in March, the rain regime average was ranging from 4.2–216.9 millimeters per month with a monsoon period from June to October and the relative humidity was ranging from 45% to 77% (data collected from weather station of the Thai Meteorological Department—Chiang Mai). The temple is abandoned and located in a residential area (Figures 1A,B). There is a *Samanea saman* (Jacq.) Merr. tree (*Fabaceae*) at the western side of the pagoda and below ground pagoda is surrounded by round eggplants [*Solanum melongena* L. (*Solanaceae*)] and Siam weeds [*Chromolaena odorata* (L.) R.M.King & H.Rob. (*Asteraceae*)]. The mural painting was discovered in 2019 in the inner part of a pagoda tunnel, 55 cm in width, 120 cm in height and 140 cm long. The mural was painted on walls at the end and on both sides of the tunnel (Figure 1C). It was painted using the fresco technique, water-based pigment on fresh, fine-grained plaster (Piovesan et al., 2012), which was commonly used during that time. Red and black were used as the primary colors of the painting (Figure 1C). The painting was fading, thus, to present the clear picture of the painting, we copy the mural painting line on transparent paper with 1:1 scale and then adjust it on Adobe Illustrator Cs6 (Figure 1D).

### 2.2. Powder diffraction (PXRD) analysis of painting colors and background material

Powder X-ray diffraction (PXRD) (Rigaku SmartLab, Cu Kα, λ = 1.541 84 Å) was used to identify the crystalline phases in the well-grounded samples. The solid samples were collected and grounded in an agate mortar. The XRD patterns were collected in the range of 2θ = 10–60°, and then matched and indexed carefully using X’pert High Score Plus program. The PXRD patterns were drawn by Origin program.

### 2.3. Sample collection

Samples were taken in August 2020 using aseptic procedures. Six square plots (10 cm^2^) were randomly selected as representative samples from the painting. Three subsamples were collected from each plot using sterile swabs. The swabs from the same sampling points were collected in the same tube to make a composite sample. The composite samples were stored in DNA/RNA Shield (Zymo Research, Irvine, CA, United States) for microbial community analysis using a culture-independent method, and in 0.1% Tween 80 for microbial isolation using culture plates (Suphaphimol et al., 2022). At the sampling time, the temperature in the main hall was 29.8 ± 0.2°C with a relative humidity of 46.2% ± 2.4% and a light concentration of 8.4 ± 0.3 Lux. However, the painting surface was dry, due to the location of the painting deep in the tunnel, and the tunnel faces to the eastern side (Raining wind in Thailand normally flows from the southern part). Since algae did not appear on the painting, we omit algae from the investigation.

### 2.4. Microbial characterization: community, functional and network analysis using high-throughput sequencing

DNA was extracted from all samples collected from the mural paintings, using Quick-DNA Fecal/soil Microbe Microprep kit (Zymo Research, Irvine, CA, United States) following the manufacturer’s protocols. The DNA was then amplified on V3 – V4 region of 16S rRNA gene, using primer 341F (5’-CCTACGGGNGGCWGCAG-3′) and 805R (5’-GACTACHVGGGTA-TCTAATCC-3′), for both archaeal and bacterial study (Klindworth et al., 2013; Wasimuddin Schlaeppi et al., 2020) and ITS1 regions, using ITS1F (5’-CTTGGTCATTTAGGAAGTAA-3′) and ITS2R (5’-GCTGCGTTCTTCATCGATGC-3′), for fungal study (White et al., 1990). The amplicon was then sequenced using Illumina MiSeq platform (2 × 300 bp). The amplification and sequencing analysis were processed by Macrogen (Seoul, Korea). The raw sequence data of 16S rRNA and ITS gene were deposited in the National Center for Biotechnology Information (NCBI) under the accession number PRJNA838707.

The sequencing data were analyzed on QIIME2 software (Bolyen et al., 2019). Firstly, prokaryotic and fungal primers were removed using Cutadapt (Martin, 2011). The trimmed sequences were then quality-filtered, merged, and chimeras removed using DADA2 (Callahan et al., 2016). Amplicon sequence variants (ASVs) that contained less than 2 sequences were removed from the dataset to eliminate potential sequencing errors. The remaining data were taxonomically classified with the Silva database version 138 (Pruesse et al., 2007; Quast et al., 2013) for bacteria and UNITE database version 8.0 (Abarenkov et al., 2021) for fungi. Bacterial and fungal diversity indexes, including observed richness, Shannon and Simpson, were analyzed with “phyloseq” as implemented in R software version 4.2.2 (R Development Core, 2019).

The community data of microorganisms were subjected to a correlation analysis using the “Hmisc” package (Harrell and Dupont, 2023) in R software version 4.2.2., employing a correlation coefficient threshold of 0.8 and a significance level of 0.05. The resulting correlation coefficient data were visualized using Cytoscape version 3.9.1.

### 2.5. Microbial isolation and their potential survival in dry environment

#### 2.5.1. Isolation and identification of culturable microbes

Samples collected for the culture-dependent method were shaken thoroughly and serially diluted by 0.85% (*v*/*v*) of NaCl to get the concentration ranging from 10^−1^ to 10^−3^. After that, 100 μL of each concentration was inoculated on tryptic soy agar (TSA) plates and potato dextrose agar (PDA) plates, for bacterial and fungal isolation, respectively. The culture plates were incubated at 30°C, under aseptic condition, for 24–72 h for bacteria and 3–5 d for fungi. Colonies showing different morphology were selected and transferred to fresh plates to obtain pure isolates.

Genomic DNA was extracted from bacterial and fungal pure isolates using PureLink Genomic DNA mini Kit (Invitrogen, Walthan, MA, United States), following the manufacture protocol. Specifically, the fungal pure cultures were prepared following the thermolysis method (Zhang et al., 2010) before subjected to DNA extraction kits. Briefly, fungal mycelia on a culture media were transferred into pure water in microcentrifuge tube, vortexed thoroughly, and centrifuged. The supernatant was then discarded and the remaining part was used for DNA extraction (Zhang et al., 2010). Bacterial and fungal DNA were amplified through PCR using the 27F (5’-AGAGTTTGATCMTGGCTCAG-3′) and 1492R (5’-TACGGYTACCTTGTTACGACT-3′) primer set for bacteria and the ITS5 (5′-GGAAGTAAAAGTCGT AACAAGG-3′) and ITS4 (5’-TCCTCCGCTTATTGATATGC-3′) primer set for fungi (Suphaphimol et al., 2022). The PCR conditions were used as previously described by Suphaphimol et al. (2022). All PCR products were sequenced with dideoxy method by Macrogen (Macrogen Inc., Seoul, Korea). The obtained sequences were then edited and aligned using Bioedit (version 7.1.1) (Hall, 1999). Taxonomic identification of each isolate was performed by comparing the sequences to those in the NCBI database using BLASTn. All sequences derived in this study were submitted to NCBI under the accession number as presented in Supplementary Table S1.

#### 2.5.2. Viability of microorganisms under dry/drought conditions

The ability of the isolates to survive under dry/drought conditions, which are the prevailing conditions in the pagoda tunnel, was tested using the sorbitol method (Hallsworth et al., 1998; Lasudee et al., 2018). Briefly, TSA and PDA media were prepared by mixing the media components with 0, 175, 285, 405, 520, 660, and 780 g/L of sorbitol to adjust the water activity of the media to 0.998, 0.976, 0.957, 0.919, 0.844, and 0.807, respectively. The microbial isolates were then inoculated onto the media. Isolates that grew on media with 0.919 a_w_ or lower were considered drought-tolerant (Lasudee et al., 2018).

### 2.6. Evaluation of biodeterioration potentials of bacterial and fungal isolates

#### 2.6.1. Discoloration

In this study, we used ferric oxide calcined and charcoal activated as the representatives of red and black colors found in the study site. The ability of microbial isolates to potentially decolorate colors on the mural painting were investigated using a modified method from Suphaphimol et al. (2022). In detail, 0.8% (w/v) of the colors were separately suspended in culture media. For bacteria, discoloration activities were tested by the well diffusion approach. Bacterial isolates were incubated in tryptic soy broth (TSB) for 24 h, and then the isolates were adjusted to 1.0 McFarland standard and placed in the agar well of TSA mixed with the tested colors. On the other hand, fungal isolates were incubated on PDA for 24 h, then harvested using a 7 mm cock borer and placed on a PDA plate mixed with the tested colors. Both bacterial and fungal isolates were incubated for about 3 and 5 d, respectively. An isolate producing a halo zone was considered as a potential isolate to decolorize the painting.

#### 2.6.2. Solubilization of insoluble minerals (calcium compounds)

Since the main component of the mural wall is calcium compounds, in this study we test the ability of microbial isolates to solubilize insoluble calcium compounds. The solubilizations were determined using methods described by Fomina et al. (2005) and Kumla et al. (2014). Briefly, basal media were individually mixed with 0.3% (w/v) of calcium carbonate (CaCO_3_), calcium phosphate [Ca_3_(PO_4_)_2_], zinc carbonate (ZnCO_3_), feldspar, and kaolinite. Bacterial and fungal isolates were placed on the prepared media as described in 2.5.1 and incubated in darkness for 3 d (bacteria) and 5 d (fungi). Isolate producing a solubilization zone (halo zone) is considered as a potential isolate to degrade the painting layers.

#### 2.6.3. Mineralization plate assay evaluating the ability to produce calcium compounds

Each of the isolates underwent testing to determine its capacity to induce the precipitation of calcium carbonate using the procedure described by Ma et al. (2020). Briefly, microbial isolates were incubated in a modified B4 medium, which included calcium acetate [(CH_3_COO)_2_Ca]. The incubated substrates were fragmented into smaller specimens and subsequently subjected to desiccation within a heated incubation apparatus for 72 h, prior to being coated with a layer of gold for investigating the formation of calcium crystals. Calcium precipitates were observed using a scanning electron microscope, JSM 5910 (JEOL, Akishima, Tokyo, Japan).

## 3. Results

### 3.1. Chemical composition of painting colors and background revealed by powder diffraction (PXRD) analysis

**Figure 2:**
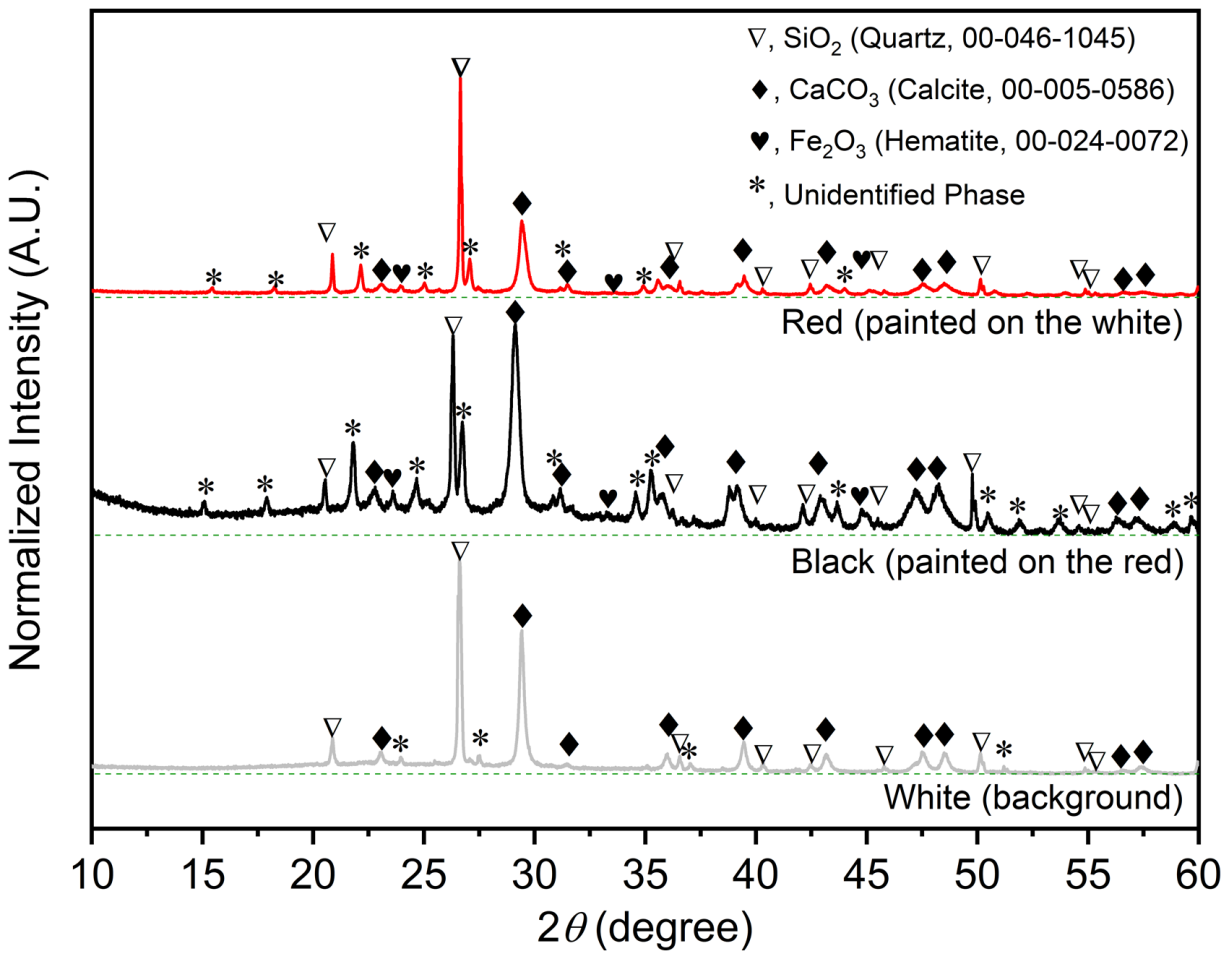
Indexed PXRD patterns with baselines (dashed green line) of the bulk solid samples of the wall-painting materials sampled from the three different areas.

The PXRD is non-destructive and useful tool for phase identification and analyzing functional groups and chemical components in materials. The well-indexed PXRD patterns of the three different colored samples (Figure 2); (i) the white (background), (ii) the red, and (iii) the black; unambiguously reveal that the common and major construction materials are sand (Quartz, SiO_2_, 00–046-1,045) and limestone (Calcite, CaCO_3_, 00–005-0586). The colorless (white) SiO_2_ and CaCO_3_ were fine and commonly used as painting background materials. The characteristic peaks of Hematite (Fe_2_O_3_), the red painting materials, and some unidentified crystalline phases can be observed in PXRD patterns of the red and black samples. Note that the significantly high background in the PXRD pattern of the black sample indicates the amorphous phase composition, which could be both the organic and other carbonous painting materials.

### 3.2. Overview of sequencing information and alpha diversity

Overall, a total of 417,264 reads, assigned to 205 prokaryotic ASVs (35 archaeal ASVs and 270 bacterial ASVs) and 631,710 reads, assigned 140 fungal ASVs, were obtained in this study. At the analysis sequencing depth, the rarefaction curves were gradually flattened, indicating that the obtained reads were sufficient to represent all microbes in the communities (Supplementary Figure S1). As shown in Table 1, bacteria are more diverse than fungi and archaea, accounting for two and three times higher than archaea and fungi, respectively.

**Table 1:** Alpha diversity indexes of microbes observed in Lan Na’s mural painting.

Samples ID	Observed Richness			Shannon’s Index			Simpson’s index		
	Archaea	Bacteria	Fungi	Archaea	Bacteria	Fungi	Archaea	Bacteria	Fungi
No. 1	18	38	13	0.8234	1.918	0.0747	0.3049	0.7700	0.0204
No. 2	17	54	21	0.8709	2.129	0.0639	0.3412	0.8016	0.0165
No. 3	18	47	25	0.8059	2.068	0.1122	0.3068	0.8132	0.0296
No. 4	18	48	25	0.9191	1.683	0.1056	0.3567	0.6813	0.0274
No. 5	20	83	95	0.9432	2.118	0.5881	0.3646	0.7928	0.3275
No. 6	24	116	42	0.8505	2.504	0.2900	0.3119	0.8481	0.1261
Mean ± SE	19.17 ± 1.07	64.33 ± 12.19	36.83 ± 12.26	0.8688 ± 0.0219	2.07 ± 0.1106	0.2059 ± 0.0836	0.3310 ± 0.0108	0.7845 ± 0.0232	0.0912 ± 0.0502
Total ASVs	35	270	140						

### 3.3. Taxonomic distribution of microorganisms associated with the mural painting

**Figure 3:**
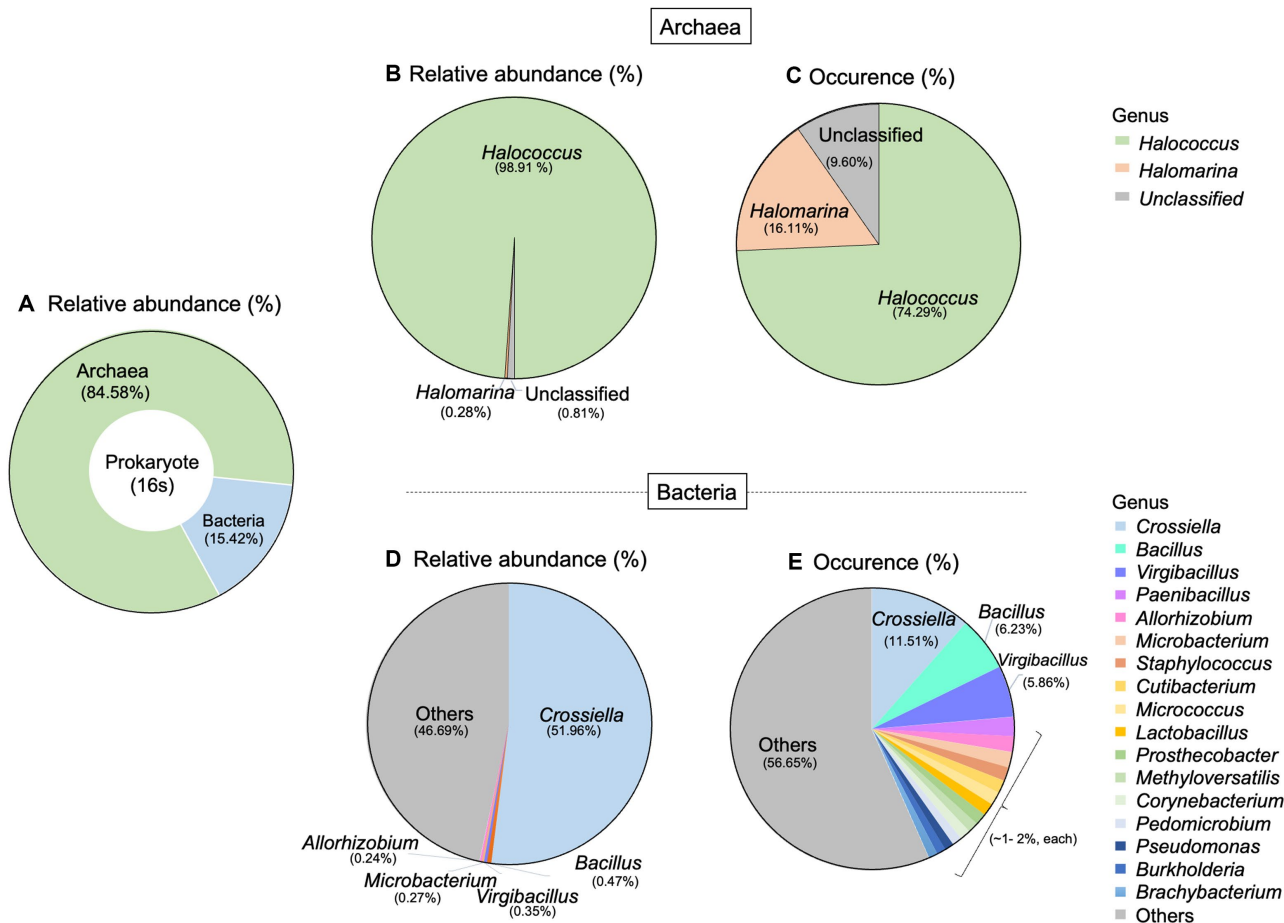
Taxonomic distribution of prokaryote associated with Lan Na’s mural painting. **(A)** The proportion of archaea and bacteria found in this study. **(B)** Relative abundance and **(C)** occurrence of archaea genera. **(D)** Relative abundance and **(E)** occurrence of bacterial genera. These data are average proportions across all samples.

For prokaryotic taxa, the relative abundance of archaea was much higher than that of bacteria, accounting for 84.58% (Figure 3A). Here, we detected two archaeal genera which are *Halococcus* and *Halomarina*. These two genera belonged to the phylum *Euryarchaeota*, Class *Halobacteria*, Order *Halobacteriales*, and Family *Halococcaceae*. The relative abundance and occurrence percentage of *Halococcus* are much higher than those of *Halomarina,* with more than 98% of relative abundance and about 74% of occurrence (Figures 3B,C). More diverse taxa were found in bacteria, compared to archaea. Fourteen phyla, 23 Classes, 50 orders and 106 genera were derived in this study. The most abundant phyla belonged to *Actinomycetota* (*Actinobacteria*), followed by *Bacillota* (*Firmicutes*), and *Pseudomonadota* (*Proteobacteria*). At the genus level, *Crossiella* obtained about 50% of total relative abundance and the occurrence percentage of this genus was 11.51%. Whilst *Bacillus* and *Virgibacillus* were found in a very low abundance (<1%), their occurrences were 6.23% and 5.86%, respectively (Figures 3D,E).

**Figure 4:**
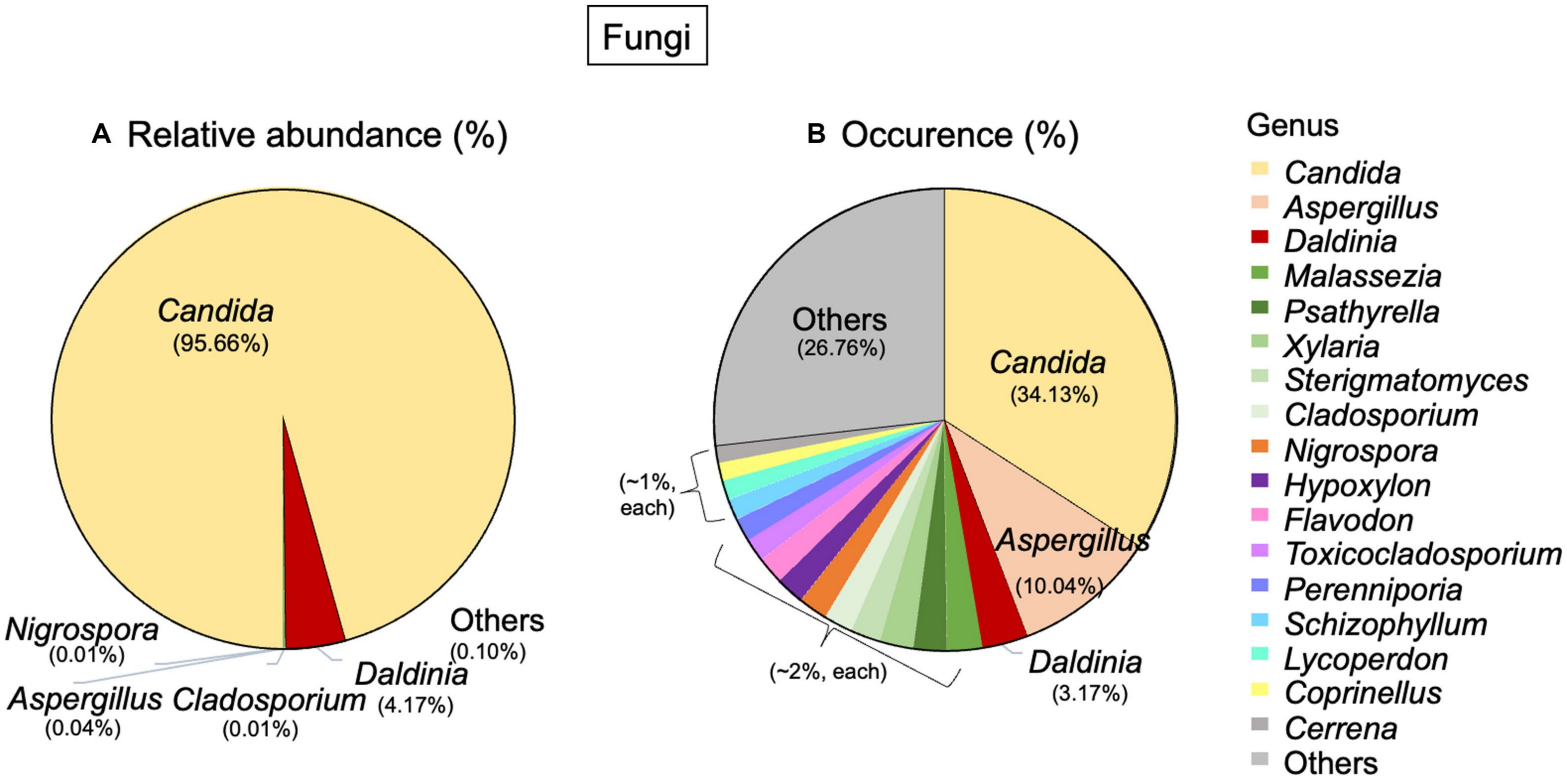
Taxonomic distribution of fungi associated with Lan Na’s mural painting. **(A)** Relative abundance and **(B)** occurrence of fungal genera. These data are average proportions across all samples.

Regarding fungi, 2 phyla, 13 classes, 25 orders, and 55 genera were detected. The most abundant phylum was *Ascomycota* (99.94%), and the other phylum was *Basidiomycota* (0.05%). At the genus level, *Candida* was the dominant genus in terms of both abundance (95.66%) and occurrence (34.13%). *Daldinia* had an abundance of 4.17% and an occurrence of 3.17%. *Aspergillus*, on the other hand, had a very low abundance, but it occurred in about 10.04% of the total fungal community (Figures 4A,B).

### 3.4. Network analysis

**Figure 5:**
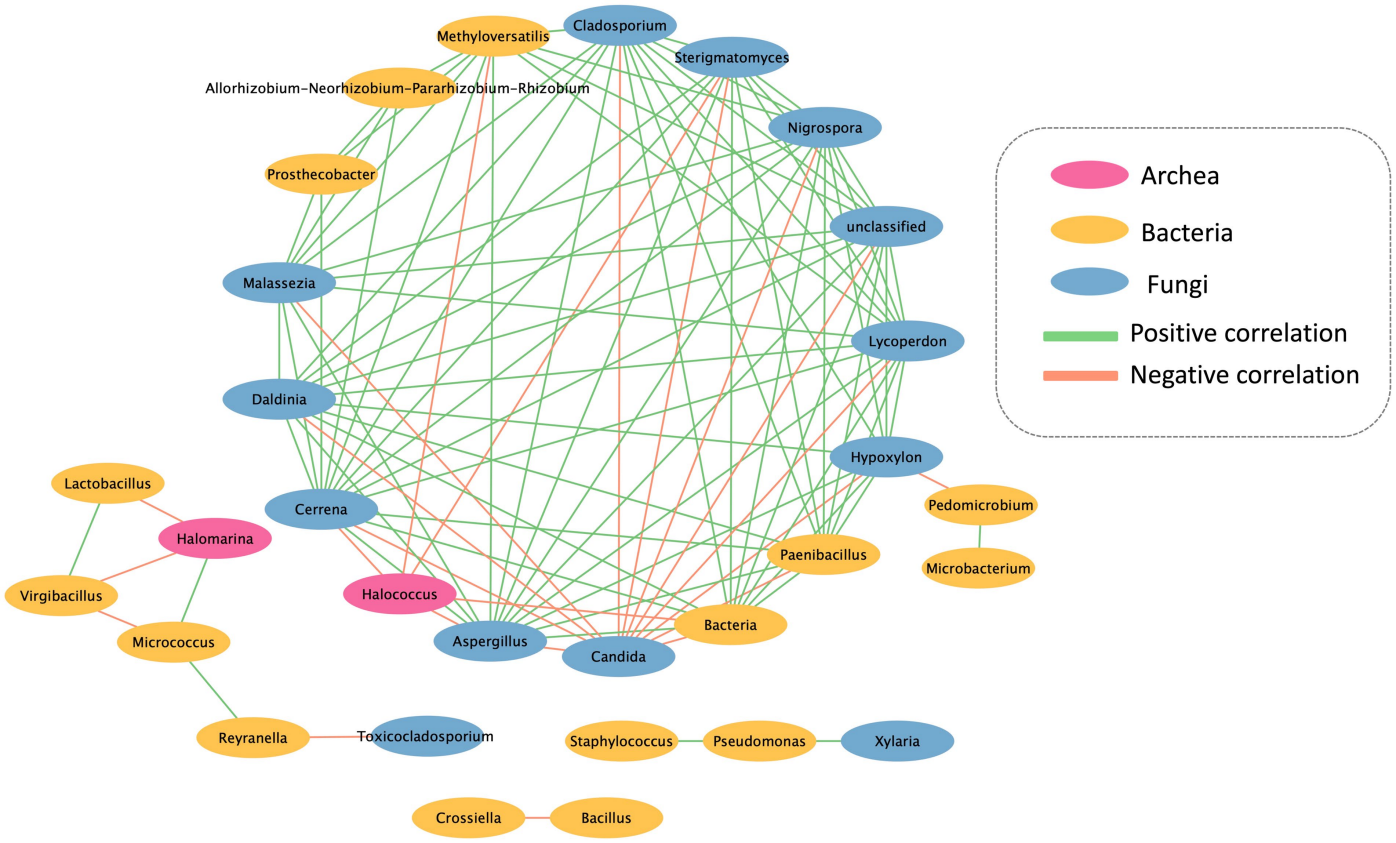
Interkingdom network between archaea, bacteria and fungi found in Lan Na’s mural painting.

The co-occurrence network analysis pattern of the microbial community present on the mural painting within the temple was examined by assessing its statistically significant correlations (Figure 5). The network exhibits both positive and negative correlations among distinct microorganism groups. The network encompasses 30 nodes and 103 edges, portraying the interactions between these microbial entities. Notably, *Candida* sp. and *Halococcus* sp. emerge as the predominant groups, displaying a noteworthy negative association with other bacterial groups. Furthermore, the network delineates the presence of three distinct sub-networks, as depicted in the accompanying figure.

### 3.5. Culturable isolates and their ability to deteriorate the painting

#### 3.5.1. Identification of the microbial isolates and their ability to survive in dry condition

Six bacterial and two fungal isolates were obtained from the mural painting samples in Sean Khan temple. As shown in Table 2, the bacterial isolates were identified as *Staphylococcus epidermidis* (SKB1, SKB2, and SKB5), *Bacillus licheniformis* (SKB3), *Bacillus amyloliquefaciens* (SKB4), and *Burkholderia cepacia* (SKB6), while the fungal isolates were identified as *Trichaptum* sp. (SKF1) and *Aspergillus* sp. (SKF2). When subjected to the isolated dry condition using the sorbital method, we found that almost all bacterial and fungal isolates, except for isolate *Trichaptum* sp. (SKF1), were able to survive in drought conditions (aw = 0.957 or higher) (Table 2). Sequences of all isolates were submitted to NCBI and the accession number of each isolate was presented in Supplementary Table S1.

**Table 2:** Molecular identification of culturable microbes and their biodeterioration potentials.

Isolate	Identification by NCBI BLASTn			Drought tolerance (survived at aw ≥0.919)	Discoloration		Calcium compound					Crystal formation
	Closest relative taxa	Similarity (%)	Accession number		Ferric oxide calcined	Charcoal activated	CaCO_3_	Ca_2_PO_4_^2−^	ZnCO_3_	Feldspar	Kaolinite	
Bacteria												
SKB1	*Staphylococcus epidermidis*	100	AB617572	✓	–	–	✓	–	✓	–	–	–
SKB2	*Staphylococcus epidermidis*	100	KJ571206	✓	–	–	–	–	✓	–	–	–
SKB3	*Bacillus licheniformis*	99.93	MK859953	✓	–	–	–	–	✓	–	–	–
SKB4	*Bacillus amyloliquefaciens*	99.79	MT579842	✓	–	–	–	–	✓	–	–	–
SKB5	*Staphylococcus epidermidis*	100	KT372242	✓	–	–	✓	–	✓	–	–	–
SKB6	*Burkholderia cepacia*	100	MZ049603	✓	–	–	✓	–	–	–	–	–
Fungi												
SKF1	*Trichaptum* sp.	100	MW661085	–	–	–	✓	✓	–	–	–	✓
SKF2	*Aspergillus* sp.	100	MT530274	✓	–	–	✓	✓	✓	–	–	–

#### 3.5.2. Discoloration, solubilization of calcium compounds

Here, we tested the ability of microbial isolates to discolor calcined ferric oxide and activated charcoal. However, no isolates showed the ability to discolor them. In contrast, several isolates were capable of solubilizing calcium compounds. Bacterial isolates *Staphylococcus epidermidis* (SKB1, SKB5), *Burkholderia cepacia* (SKB6), and fungal isolate *Trichaptum* sp. (SKF1) and *Aspergillus* sp. (SKF2) were able to solubilize calcium carbonate, while only fungal isolate *Trichaptum* sp. (SKF1) and *Aspergillus* sp. (SKF2) was able to solubilize calcium phosphate. Almost all isolates, except for *Burkholderia cepacian)* (SKB6) and *Trichaptum* sp. (SKF1), were able to solubilize zinc carbonate (Table 2).

#### 3.5.3. Identification of calcium compounds produced by microbial isolates

**Figure 6:**
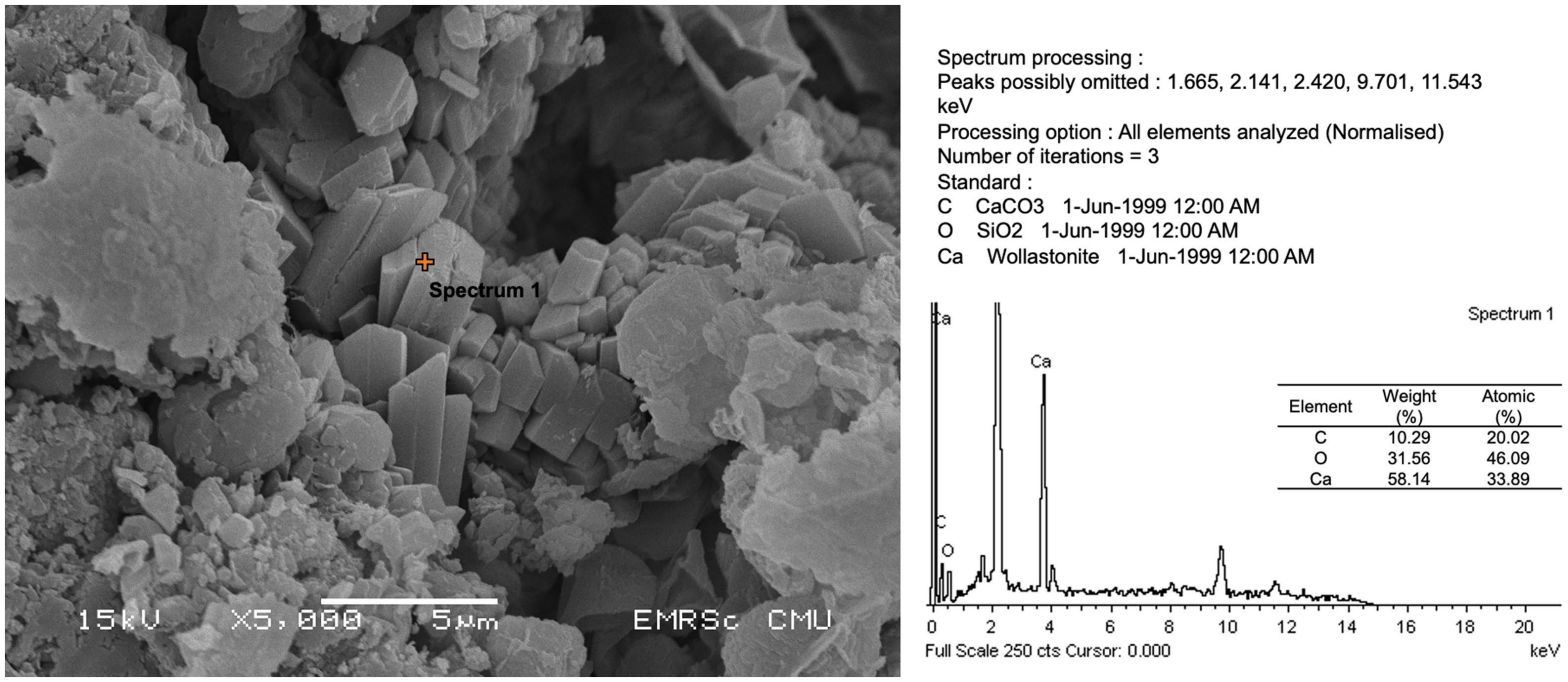
Calcium compound formation and energy-dispersive X-ray spectroscopy (EDS) analysis of isolate SKF1 (*Trichaptum* sp.).

All microbial isolates were subjected to a culture medium with calcium compound to test the ability to participate in calcium precipitation or form crystals. As shown in Table 2 and Figure 6, no bacterial isolate was able to form the crystal structure, whereas fungal isolates *Trichaptum* sp. (SKF1) could form the crystal structure.

## 4. Discussion

The study of biodeterioration activities on mural paintings has been the central focus for decades. However, in northern Thailand, where a unique cultural style called “Lan Na” developed, little research has been conducted. This study was carried out at the Sean Khan temple in Chiang Mai, Thailand and was the first study on 13th-century Lan Na mural painting. We identified microbial communities, including archaea, bacteria, and fungi, associated with the paintings and determined the possible deterioration capacity of these microbes.

For prokaryotes, more than 80% of the total prokaryotic community belonged to archaea and about 15% were bacteria. This result was contradicted to the study on limestone walls of the old cathedral of Coimbra which found more diverse bacteria than archaea (Coelho et al., 2021). In the case of our study site, a neglected mural painting, water seepage into the painting substrate can result in the formation of salt efflorescence as the water evaporates (Amoroso and Fassina, 1983; Laiz et al., 2000). The accumulation of salts creates a high salt concentration environment, which in turn selects for halophiles. Our results demonstrate a significant proportion of haloarchaea within the archaeal communities. The most prevalent taxa were *Halococcus*, with a small portion of *Halomarina*, both of which are halophilic archaea. Archaea has been identified in several deteriorated ancient wall paintings (Rölleke et al., 1998; Piñar et al., 2001). *Halococcus* is typically found colonizing ancient wall paintings and is known to be a part of bacterioruberin-producing groups, which could potentially alter the color of frescoes (Piñar et al., 2001 Ettenauer et al., 2010, 2014; Coelho et al., 2021) (Table 3). Moreover, we found that *Crossiella* dominated the bacterial community. *Crossiella* has been identified as the predominant actinobacteria associated with salt efflorescence (Lepinay et al., 2018). This finding suggests that the prokaryotic communities inhabiting this Lanna painting have adapted to the specific conditions associated with salt efflorescence. *Bacillus* and *Virgibacillus* were the major members of the bacterial community. *Bacillus* and related genera (i.e., *Paenibacillus*) were also reported on mural paintings in Germany (Gorbushina et al., 2004), Spain (Jurado et al., 2021), and also in other Lan Na’s mural paintings in Thailand (Suphaphimol et al., 2022). However, the abundance of *Bacillus* is not new, as they are ubiquitous bacteria which can be found in any environment. On the other hand, the prevalent taxa dominated fungal communities was *Candida*, followed by *Daldinia* and *Aspergillus*. *Candida* was also reported on mural painting in Japan (Sugiyama et al., 2017) and discolored walls in Nigeria (Obidi and Okekunjo, 2017). *Aspergillus*, on the other hand, was generally found in mural paintings (Suphaphimol et al., 2022).

**Table 3:** Potential biodeterioration capacity of microbial taxa detected by the culture-independent method.

Taxa detected in this study	Source and potential biodeterioration	References
Archaea		
*Halococcus*	Found on rosy discolored wall painting (rosy biofilm) at the Johannes Chapel in Purgg, the castle Rappottenstein and the Saint Rupert Chapel in Wei preach.; limestone wall of the Old Cathedral of Coimbra (Portugal) Unculture; No biochemical test on biodeterioration capacity. Some culturable Halophilic microorganisms potentially discolored rosy color. Found on valuable mural painting at the Catherine Chapel of the Castle Herberstein, Austria and the mural painting from Roman Necropolis of Carmona, Spain.	Piñar et al. (2001), Ettenauer et al. (2014), and Coelho et al. (2021)
Bacteria		
*Arthrobacter*	*Arthrobacter agilis* found on *Terme del Foro (Pompeii, Italy). Six novel Arthrobacter sp. found on wall painting at* Catherine Chapel of the Castle Herberstein, Austria and the mural painting from Roman Necropolis of Carmona, Spain. *Arthrobacter aurescens found on medieval wall painting of the shurch of St. Georgen, (Styria, Austria).* *Mural painting of St. Martins church (Germany)* Rosy discolorations.	Gorbushina et al. (2004), Heyrman et al. (2005b), and Tescari et al. (2018)
*Crossiella*	Isolate from biofilm on cave wall (Altamira Cave, Spain). Found in limestone wall of the Old Cathedral of Coimbra (Portugal) Fresco painting, Cave Church of the Sts.Peter and Paul (Serbia) Bioactive compounds inhibit the growth of other microorganisms.	Coelho et al. (2021), Gonzalez-Pimentel et al. (2022), and Dimkić et al. (2023)
*Bacillus*	Mural painting of St. Martins church (Germany) Deteriorate wall painting sample by calcium precipitation. *Bacillus* sp. found on deteriorated mural painting at Necropolis of Carmona, Spain.	Gorbushina et al. (2004), Heyrman et al. (2005a), Silva et al. (2015), and Helmi et al. (2016)
*Paenibacillus*	Mural paintings in the Servilia tomb. Mural painting of St. Martins church (Germany) Acid production.	Gorbushina et al. (2004) and Šmerda et al. (2006)
*Virgibacillus*	Found on biofilm formation on the mural paintings of the Servilia tomb (Spain) and the Saint-Catherine chapel (Austria).	Heyrman et al. (2003)
*Staphylococcus*	Found on Lan Na mural painting (Thailand) and an ancient easel painting (Italy) Biofilms of an old building in Pune (India)	Jadhav et al. (2010), Caselli et al. (2018), and Suphaphimol et al. (2022)
*Pseudomonas*	Fresco painting, Cave Church of the Sts. Peter and Paul (Serbia) Deteriorating painted wall surfaces in Lagos (Nigeria)	Obidi et al. (2022) and Dimkić et al. (2023)
Fungi		
*Aspergillus*	Adherents to the mural surface. Mural painting of St. Martins church (Germany) Fade the paint color and break the paint surface. Acid formation and calcium compound precipitation.	Gorbushina et al. (2004), ISHFAQ et al. (2015), and Suphaphimol et al. (2022)
*Cladosporium*	Adherents to the surface of modern wall painting, Weyregg, and Austria. Mural painting of St. Martins church (Germany) Possibly cause staining. Enzyme secretion(cellulase, polyphenol oxidase)	Gorbushina et al. (2004), Rojas et al. (2012), and Sterflinger and Piñar (2013)
*Candida*	Mural painting in Japan and discolored walls in Nigeria Deteriorating painted wall surfaces in Lagos (Nigeria) pathogenic agent	Obidi and Okekunjo (2017), Sugiyama et al. (2017), and Obidi et al. (2022)

The interrelationships among microorganisms were investigated using network analysis. A majority of microorganisms displayed a positive correlation, indicating their potential to provide a favorable substrate and environment for the proliferation of other microorganisms. However, *Candida* sp. and *Halococcus* sp. exhibited a negative correlation with the other microorganisms. The colonization of *Candida* sp. was found to inhibit the growth of other microorganisms due to the production and secretion of proteinase or phospholipase enzymes, which can impact other organisms negatively (Pannanusorn et al., 2020). Furthermore, *Halococcus* sp. demonstrated the ability to generate halocins, which are protein-based antimicrobial substances. These halocins function by inhibiting the Na^+^ and H^+^ antiporter, leading to cell lysis and ultimately preventing the growth of other microorganisms (Kumar et al., 2021). However, *Halomarina* sp. exhibits a positive correlation with *Micrococcus* sp., while displaying a negative correlation with the remaining bacterial groups. Previous research indicates that both *Microoccus* sp. and *Halomarina* sp. possess the capability to synthesize carotenoids (Galiana et al., 2022), which may serve as antimicrobial agents against the other bacterial groups. Consequently, this leads to a negative correlation with *Lactobacillus* sp. and *Virgibacillus* sp. (Vargas-Sinisterra and Ramírez-Castrillón, 2021).

As shown in Table 3, *Crossiella* promotes calcite formation in caves and is very abundant in moonmilk (Martin-Pozas et al., 2022). Furthermore, it was reported that this genus potentially inhibits the growth of several bacteria and fungi such as *Bacillus cereus*, *Staphylococcus aureus*, *Aspergillus versicolor*, *Penicillium chrysogenum*, and *Fusarium solani* (Gonzalez-Pimentel et al., 2022). The abundance of this taxa may inhibit the growth of several taxa on the painting surface. Although the biodeterioration potentials of *Candida* has not been studied, several members of this genus were presented as pathogenic agent (Turner and Butler, 2014; Obidi and Okekunjo, 2017). It was suggested that the presence of this group with a large proportion might be from animals, insects and their fecal pellets and may be influenced by the presence of visitors, which contribute to the movement of airborne particles (Sugiyama et al., 2017; Zucconi et al., 2022). *Aspergillus*, on the other hand, showed biodeterioration activities. For example, *Aspergillus fumigatus* and *Aspergillus piperis* were able to produce acid and calcium compounds which potentially deteriorate the mural paintings (Suphaphimol et al., 2022). However, it should be noted that these are the predictive metabolic potential of microbial communities found in this study. These data provided only a general assessment. Empirical activity measurements are necessary for a more accurate representation. Conducting activity assessments through enrichment or co-culture approaches would allow for a closer capture of the true metabolic potential of microbial communities.

Culture dependent method could provide the information on biodeterioration potentials. The isolated cultures can be used for further investigation of the processes and mechanisms causing the biodeterioration of mural paintings. In this study, six bacteria and two fungal isolates were obtained and were identified as *Staphylococcus epidermidis*, *Bacillus licheniformis*, *B. amyloliquefaciens*, *Burkholderia cepacia, Trichaptum* sp., and *Aspergillus* sp. *Staphylococcus* sp. and *Aspergillus* sp. were found in the mural painting in many studies (Jadhav et al., 2010; Caselli et al., 2018; Suphaphimol et al., 2022; Zucconi et al., 2022). *Burkholderia cepacia* was reported in soil or water samples (Miller et al., 2002), while *Trichaptum* sp. was found in the environment as a saprophytic fungus (Gafforov et al., 2020). As compared to previous study, some isolates found in this study were different from those on another Lan Na mural painting in the same region (Suphaphimol et al., 2022). These may be caused by different abiotic factors, such as temperature, relative humidity and light radiation, and different mural materials. However, notice should be taken that small number of microbes were isolated and no halophilic archaea was cultured. This may be caused by the fact that only two culture media were used in this study. To captured more diverse microorganisms, diverse type of culture media should be used in further study. Specifically, studies on murals that have undergone severe deterioration should incorporate selective culture media, particularly those with high salt content, to enhance the isolation efforts.

The mural painting in this study was created using the fresco technique. The based materials are composed of SiO_2_ and CaCO_3_, whereas red color and black colors are hematite (Fe_2_O_3_) and carbonous painting materials, respectively. Although, almost all isolates were identified as drought stress tolerant microbes which were able to growth and survive in dry condition of the temple, no discoloration activities were found in all cultured isolates. Discoloration of this painting may be caused by the uncultured microbes, such as the halophilic archaea, other abiotic factors or the deterioration of the based materials. Here, we found that five out of eight isolates, including SKB1 (*Staphylococcus epidermidis*), SKB5 (*S. epidermidis*), SKB6 (*Burkholderia cepacia*), SKF1 (*Trichaptum* sp.), and SKF2 (*Aspergillus* sp.), were able to dissolve CaCO_3_, which found to be the base material of the Lan Na’s mural painting in this study. The dissolution of this calcium compound may result from secondary metabolite secreting from the microorganism which could cause severe damage to the paint layers (Čukovska et al., 2017). We further found that isolate SK1, *Trichaptum* sp., was able to form calcium crystals which could further crack the paint layer and deteriorate the painting. The calcium crystal formation is one of the factors that generally damage or stain to the mural painting (Pérez-Alonso et al., 2004; Nevin et al., 2008). Although, previous studies showed that *Bacillus licheniformis* can precipitate the calcium compound and several *Bacillus* species capable of carbonatogenesis which is useful for stone restoration (Vahabi et al., 2013; Jroundi et al., 2017). Our results show that *Bacillus licheniformis* and *B. amyloliquefaciens* did not have the ability to precipitate calcium carbonate and form calcium compound crystals. This may be cause by the fact that our isolation strategy may not have specifically targeted the strains of *Bacillus* capable of carbonatogenesis, which is why we were unable to find any isolates with such ability. In addition, we noticed that several microorganisms, especially bacteria (i.e., *Staphylococcus* spp. and *Burkholderia* sp.) and fungi (i.e., *Trichaptum* sp.), detected as biodeterioration agents in this study were found in low abundance (rare taxa). However, when the environmental factors changes, these rare taxa could be highly active and destroy the painting (Ciferri, 1999; Martin-Pozas et al., 2022). Thus far, avoiding the favorable conditions of these rare taxa would be recommended in order to preserve the painting. We suggest that future study should include the effects of environmental changes to monitor changes in the microbial community composition.

Here, we showed that the substrate components deterioration should be taken into consideration in addition to color deterioration. However, we noted that this study investigated some biodeterioration capacity and did not cover all possible biodeterioration capacity. Further study is needed to investigate on other aspects, especially those involved in biodegradation of binders, as Wu et al. (2022) presented that biodegradation of organic binders that had been used in paintings can be easily attacked by microorganisms and may lead to loss of pigment layers. Thus, binders’s deterioration should be taken into account for the study of biodeterioration of painting in future work.

This study is the first to report on the microbiome associated with a 13^th^-century Lan Na mural painting in Sean Khan Temple. Halophilic archaea, especially those belonging to *Halococcus* dominated the prokaryotic community whereas *Candida* dominated the fungal community. Based on the literature, these dominant species may play an important role in discoloration and suppression of other microorganisms. When it comes to cultural collections, no cultured isolates were found as discoloration agents. However, some of them were able to dissolve calcium carbonate, the base material of the painting (*Staphylococcus epidermidis*, *Burkholderia cepacia*, and *Aspergillus* sp.), and form the crystal of calcium compound (*Trichaptum* sp.) which could impact the deterioration of the painting. To preserve this mural painting, further study is needed to find a possible way to eliminate those microbial taxa reported as biodeterioration agents. Once the biodeterioration agents have been identified, it is necessary to evaluate their *in vitro* inhibition to control these microbes. This evaluation will then enable suggestions for future large-scale applications.

## Data availability statement

The datasets presented in this study can be found in online repositories. The names of the repository/repositories and accession number(s) can be found at: https://www.ncbi.nlm.nih.gov/, PRJNA838707.

## Author contributions

TD substantial contributions to the conception or design of the work. TD and WP supervised and received funding. TD and NSuw collected the samples. NSup, PN, TR, SY, and NSe performed the laboratory work. CS and NSup analyzed the results and wrote the original draft preparation. CS visualized data and performed bioinformatic analysis. SY, NSe, TK, NSuw, WP, and TD reviewed and edited the manuscript. All authors contributed to the article and approved the submitted version.

## Funding

Open Access funding was enabled and organized by the Department of Soil Ecology, UFZ-Helmholtz Centre for Environmental Research.

## Conflict of interest

The authors declare that the research was conducted in the absence of any commercial or financial relationships that could be construed as a potential conflict of interest.

## Publisher’s note

All claims expressed in this article are solely those of the authors and do not necessarily represent those of their affiliated organizations, or those of the publisher, the editors and the reviewers. Any product that may be evaluated in this article, or claim that may be made by its manufacturer, is not guaranteed or endorsed by the publisher.
